# A suture in time: The ontogeny of cranial suture morphology in mammals

**DOI:** 10.1111/joa.70035

**Published:** 2025-08-25

**Authors:** Heather E. White, Marco Camaiti, Abigail S. Tucker, Akinobu Watanabe, Anjali Goswami

**Affiliations:** ^1^ Department of Biological Sciences Natural History Museum London UK; ^2^ Centre for Craniofacial and Regenerative Biology King's College London London UK; ^3^ New York Institute of Technology College of Osteopathic Medicine Old Westbury New York USA; ^4^ Division of Paleontology American Museum of Natural History New York New York USA

**Keywords:** complexity, mammal, morphometrics, ontogeny, skull, suture

## Abstract

Suture shape and complexity are thought to influence skull function in mammals, supporting the evolution of ecological and morphological diversity. These aspects of suture morphology are seldom studied in a comparative context, especially relative to the multitude of comparative studies of cranial shape. Using a three‐dimensional comparative ontogenetic dataset spanning 22 species across the phylogenetic breadth of Mammalia and sampling from foetal to adult stages, we applied 3D geometric morphometrics and 2D complexity metrics to track the evolutionary and developmental morphology of three cranial sutures (interfrontal, sagittal and coronal). Shape and complexity vary across the three sutures, with complexity decreasing through ontogenetic stages for antero‐posterior sutures (interfrontal and sagittal) but showing a postnatal increase for transversal sutures (the coronal). This suggests that aging is the strongest influence on longitudinal suture complexity because of simplification and obliteration for sutures subject to tensile stresses. This adulthood trend can be explained by a necessity to consolidate the skull through fusion, coupled with the disappearing need to accommodate further brain growth. Transversally positioned sutures oppose the trend as they are subject to the compressive stresses of cranial mechanics. Additionally, our findings refute the hypothesis that placental mammals have more complex and variable sutures than marsupials reflecting their more disparate ecologies. Rather, developmental history was found to be the greatest influence on suture complexity and variability. As a result, the extreme altriciality of marsupials, and its related longer postnatal brain growth, allows them to match and surpass the suture variability found in most placentals, reaching levels otherwise found mainly in primates.

## Graphical abstract

Mammal cranial sutures are important indicators of the biomechanical and developmental pressures acting upon the skull. Across three prominent sutures dividing the vault of the mammalian skull, divergent patterns emerge both taxonomically and developmentally. Despite their different developmental strategies and evolutionary histories, marsupials and placentals display comparable disparity in suture shape. The developmental trajectories of suture complexity indicate a consistent increase until adulthood, where paired and unpaired sutures display different trends. The complexity of unpaired sutures like the sagittal and interfrontal plateaus in adulthood, while the paired ones like the coronal keep increasing their complexity, pointing towards differential reactions to biomechanical stresses in the skull.

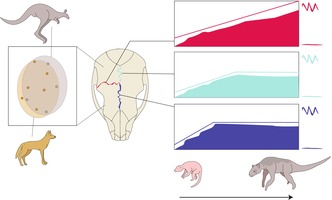

## 1 INTRODUCTION

Much research has been focused on the morphology of the mammalian skull, including its diversity, development, evolution and function. Markedly less work has explicitly considered the cranial sutures, the fibrous joints between bones, which have a critical role in shaping skulls via interstitial bone growth (Baer, 1954; Lana‐Elola et al., 2007; Opperman, 2000). Mammals display clear morphological variation across sutures (Buezas et al., 2017; Byron, 2009; White et al., 2021, 2025), but much less attention has focused on the study of their morphology and development compared to that of cranial morphology. The understanding of evolutionary and developmental patterns in mammal sutures is largely limited to analyses of individual species (Byron et al., 2004; Jaslow, 1989; Nicolay & Vaders, 2006; Sun et al., 2004), a single genus (Byron, 2009) or a single infraorder (Buezas et al., 2017). Even fewer studies consider extinct forms (but see Kammerer's, 2021 study of Permo‐Triassic dicynodonts). Moreover, only a few studies (i.e. Bieraugle et al., 2025; Nicolay & Vaders, 2006; Shibazaki et al., 2007; Sun et al., 2004) have examined changes in suture morphology through ontogenetic stages, and mostly in model organisms (respectively, miniature pigs, white deer, rats, dogs and wolves). As these studies are still limited to single species, to our knowledge no comprehensive work currently exists on interspecific sutural variation through ontogeny in mammals.

Studies that examine sutures across multiple taxa (e.g. Blumer et al., 2025; Buezas et al., 2017; Byron, 2009; Byron et al., 2023; Long & Long, 1992; Russell & Thomason, 1993) show that, even within individual genera (e.g. Cebus: Byron, 2009), considerable morphological variation can exist for the same suture across species. Highly variable suture morphologies have been observed across rodents (Buezas et al., 2017) and humans (Long & Long, 1992). Indeed, differences in suture morphology have been attributed to several factors, including phylogeny, sexual dimorphism, fighting behaviour, age and diet (Herring & Teng, 2000; Rafferty & Herring, 1999).

The leading hypothesis to explain the differences in suture shape is that the concerted, antagonistic growth of skull bones through developmental time will lead to increases in sutural complexity, specifically in the number of interdigitations (White et al., 2025; Wu et al., 2007). According to this hypothesis, sutural complexity would peak early in adulthood, after which interdigitation is lost with the age‐related process of obliteration (Sun et al., 2004; White et al., 2025). However, this has not yet been tested in quantitative or comparative frameworks, nor has it been linked to the spectrum of developmental strategies that exist in mammals. In fact, the second major untested hypothesis is that part of the variation in suture morphologies will be predicted by the broad developmental strategies adopted by a given clade. These range from extreme altriciality, where young are born in a relatively undeveloped state and require extensive parental care to achieve maturity, to precociality, where young are born in a relatively mature state and require less postnatal development and parental care (Grand, 1992). Across mammalian subclasses, monotremes and marsupials are born in a highly altricial state, while placentals range from moderate altriciality to precociality (Maier, 1993; Nunn & Smith, 1998; Sears, 2004; Smith, 1997, 2001, 2002, 2006; Tyndale‐Biscoe & Renfree, 1987). This range of developmental strategies is reflected in the developmental timing of skull bone formation (Bennett & Goswami, 2013; Goswami et al., 2009, 2012, 2016), the ontogeny of cranial shape (White et al., 2023) and adult skull disparity (Bennett & Goswami, 2013), as well as in postcranial development and disparity (Sánchez‐Villagra, 2002; Sears, 2004; Weisbecker et al., 2008) and thus can be expected to be a major driver of variation in suture morphology across mammals. Specifically, the greater morphospace occupation in skull shapes in the placentals compared to marsupials and monotremes (Bennett & Goswami, 2013; White et al., 2023), which also correlates with greater ecological diversity, can be expected to also reflect on patterns of sutural morphology. Based on these observations, we expect the developmental trajectories of suture morphology to vary significantly between more altricial and more precocial species and clades. Moreover, we expect the range of possible morphologies to differ between these groups, for instance highlighting a divide between placentals and other mammals, with the former having access to a greater range of sutural morphologies.

This study aims to address these hypotheses by quantifying and comparing the evolution and development of cranial vault suture morphology across a sample of taxa spanning the entire phylogenetic breadth of Mammalia. As such, it represents the first study of suture morphology spanning a vertebrate class. Using the largest comparative ontogenetic shape dataset to date, with 22 representative species spanning all major mammalian subclasses and most superfamilies, we quantify sutural morphology across developmental stages and taxa, focusing on three sutures of the cranial vault—coronal, sagittal and interfrontal. We focus on these sutures because of their importance in defining distinct functional and developmental domains in the skull (Goswami, 2006). The interfrontal forms between the two neural crest‐derived frontal bones, absorbing the greater biomechanical stresses this region is subjected to (i.e. mastication; Herring & Teng, 2000; Rafferty & Herring, 1999; White et al., 2021); the sagittal between the two mesoderm‐derived parietal bones, playing a critical role in supporting brain expansion and/or braincase integrity at varying developmental stages (White et al., 2021); and the paired coronal sutures at the horizontal boundary between the frontal and parietal bones (Goswami, 2006; Morriss‐Kay, 2001; White et al., 2021).

To quantify suture morphology, we take two approaches. First, we use a newly codified method of 2D projection from 3D geometric morphometrics to extract suture shape data for these three sutures. Second, we apply a series of indices to measure suture complexity, broadly defined as the minimum number of simple geometric primitives by which a structure can be represented (Gardiner et al., 2018; Orbach et al., 2020; White et al., 2020). A recent assessment of techniques to quantify suture morphology by White et al. (2020) showed that these two approaches provide a more complete picture of distinct aspects of suture morphology: shape and complexity. With this multifaceted approach, we (1) assess phylogenetic and ontogenetic shape variation across these cranial sutures; (2) quantify ontogenetic trends in suture complexity and test the hypothesis that suture complexity and disparity increase with developmental time; and (3) establish whether developmental mode is associated with differences in complexity between placentals and marsupials.

## 2 MATERIALS AND METHODS

### 2.1 Data

#### 2.1.1 Specimens

The dataset comprises foetal to adult skulls for 22 extant species of the class Mammalia, including taxa from all infraclasses (Monotremata, Marsupialia, Placentalia), all placental superorders (Afrotheria, Xenarthra, Laurasiatheria, Eucharchontoglires) and the three most diverse marsupial orders (Didelphimorphia, Dasyuromorphia, Diprotodontia). Each species is represented by specimens of four developmental stages (foetal, infant, subadult, adult). While this sampling sets the minimum number of specimens per species at four, the total number of specimens per species varies depending on the availability of specimens within museum collections, with 165 total specimens being included in these analyses (Appendix S1A). Specimens were sourced from the Natural History Museum, London (NHMUK), the University Museum of Zoology, Cambridge (UMZC), Zoologisches Museum Berlin (ZMB), Muséum national d'Histoire naturelle, Paris (MNHN), South Australian Museum (SAM), University Museum of University of Tokyo (UMUT) and Texas Memorial Museum (TMM). Specimens were micro‐CT scanned (details of scanners used for each specimen and institutions of provenance are detailed in Appendix S1A) and reconstructed using Avizo v.9.3 (FEI, Hillsboro, Oregon, USA) to produce 3D isosurfaces. To reduce computational demands, meshes were decimated in Meshlab (Cignoni et al., 2008) and then processed in Geomagic Wrap (3D Systems) to fill holes, mirror skulls damaged on one side (damaged specimens are listed in Appendix S1B) and remove associated non‐cranial structures such as vertebrae, following the protocol of Bardua, Felice, et al. (2019). The resulting 3D meshes were used for morphometric data collection.

#### 2.1.2 Developmental data

To test the role of skull development on suture shape and complexity, for each specimen, we characterised developmental age in two ways. First, discrete developmental age categories (foetal, infant, subadult, adult) were recorded based on museum collection information. Second, we estimated a continuous age metric based on skull centroid size, with relative specimen age calculated as a percentage of the adult specimen's centroid size (following Navalón et al., 2021; White et al., 2020; Zelditch et al., 2012) Both are included in Appendix S1C. Developmental mode (super altricial, altricial, semi‐altricial, semi‐precocial, precocial; Hunt et al., 2023) and diet were characterised from species descriptions in the published literature (Appendix S1D).

#### 2.1.3 Phylogenetic corrections

To account for phylogenetic relatedness among species, a phylogeny containing all 22 species within the dataset was produced by trimming the most recent and extensive time‐calibrated phylogeny of 5911 mammalian species (Upham et al., 2019), using the ‘keep. tip’ function in the R package *ape* (Paradis et al., 2004). Phylogenetically informed analyses were conducted exclusively for the adult‐only dataset, as multiple developmental stages per species cannot be collapsed into single taxonomic units (Navalón et al., 2021).

#### 2.1.4 Suture shape

To quantify suture shape, three‐dimensional landmarks and semilandmarks were manually placed by a single assessor (HW) in Stratovan Checkpoint (Stratovan Corporation, CA, USA). Landmarks (*n* = 69) were positioned across the left side of the cranium. Landmarks were mirrored using three midline landmarks (Appendix S1E), and missing landmarks (toggled as missing in Stratovan Checkpoint and assigned an *x*, *y*, *z*‐coordinate value of 9999) were subsequently estimated using the ‘estimate.missing’ function using a thin‐plate spline method in the R package *geomorph* v. 4.0 (Adams & Otárola‐Castillo, 2013). This approach was performed prior to the mirroring or Procrustes superimposition of the complete dataset. The TPS method was selected over the regression approach as it performed better when calculating missing data in simulations and does not require such a large reference sample (Gunz et al., 2009).

**FIGURE 1:**
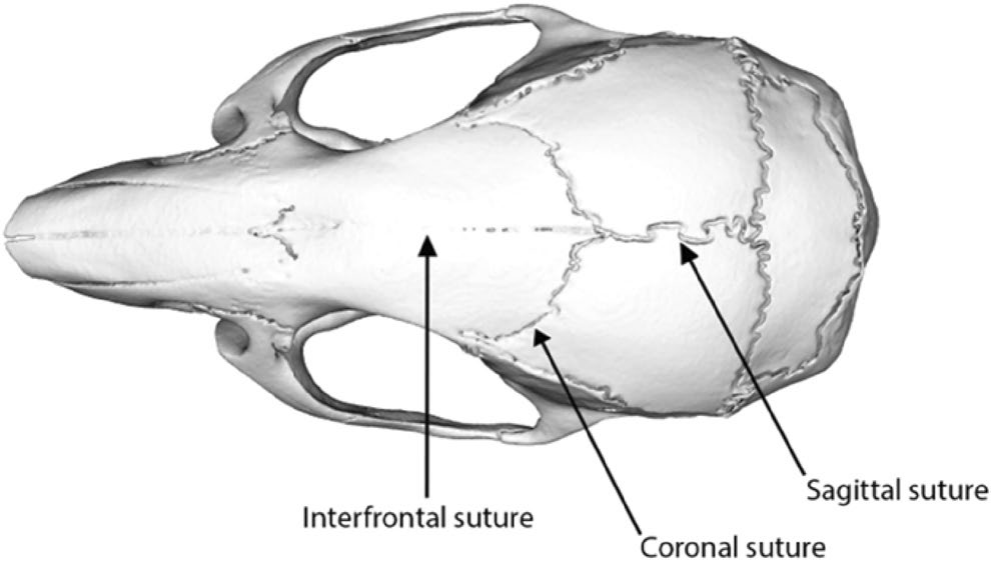
Anatomical positions of the analysed sutures, indicated on *Mus musculus*.

Semilandmarks were placed along the length of the interfrontal, sagittal and coronal sutures (Figure 1). Semilandmarks for the coronal suture, when patent, were positioned on the parietal bone side of the frontoparietal interface, as the parietal overlaps the frontal bone across the dataset, partially obscuring it in early developmental stages and complicating semilandmark placement on the frontal. To ensure even spacing and consistent numbers of semilandmarks across sutures and specimens, semilandmarks were resampled along each curve to 500 per suture (White et al., 2020). Resampled semilandmarks were then slid using the ‘slider3d’ function in the *Morpho* R package (Schlager, 2017), resulting in geometrically homologous semilandmarks (Bardua, Felice, et al., 2019; Gunz & Mitteroecker, 2013; Mitteroecker & Gunz, 2009; Schlager, 2017).

Procrustes superimposition was performed using only the original and mirrored landmarks with the ‘procSym’ function in *Morpho* (Schlager, 2017). Semilandmarks were excluded from superimposition because their high concentration in one skull region could skew Procrustes analysis (Bardua, Felice, et al., 2019; Bardua, Wilkinson, et al., 2019). Landmarks were not used in subsequent analyses, except for converting 3D semilandmarks to 2D semilandmarks for sutural complexity analysis; instead, we used the 500 sliding semilandmarks per suture, which were positioned on the left side, for a total of 1500 sliding semilandmarks per specimen.

#### 2.1.5 Suture complexity and complexity metrics

**FIGURE 2:**
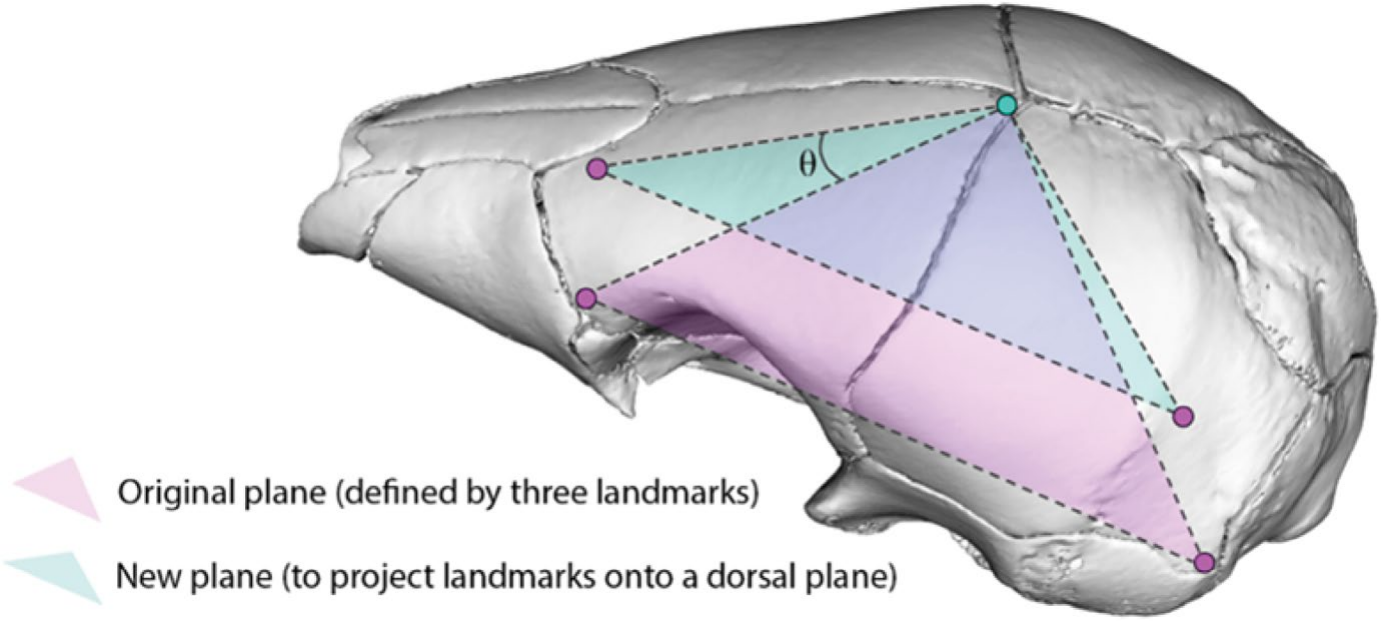
Rotation of the coronal suture sliding semilandmarks from the oblique to frontal plane, illustrated on the skull mesh of a white‐bellied pangolin (*Phataginus tricuspis*), where θ refers to the specimen‐specific angle of rotation. Dots show the landmark positions used for the calculation of the specimen‐specific angle of rotation, where the *z*‐coordinate of the pink landmarks are changed to the *z*‐coordinate of the blue landmark.

Because at present, complexity metrics are only available for 2D data, we converted all 3D geometric morphometric data for suture shape to 2D for calculating suture complexity. For the interfrontal and sagittal sutures, this was accomplished by removing *z*‐axis coordinates aligned with the specimen's dorsoventral axis, corresponding with the frontal plane, along which these sutures are located (Figure 2). For the coronal suture, which does not align consistently with any of the principal anatomical axes, three fixed skull landmarks were used to define an oblique plane that accounts for varying orientation of the skull roof. These (landmarks 20 and 23 [posteromedial most and anterolateral most points of the frontal, respectively], and landmark 27 [posteromedial most point of the parietal bone]) were selected to encompass the coronal suture within a newly defined plane. 3D semilandmarks were rotated in 3D coordinate space such that the new *z*‐axis aligned orthogonally to the orientation of the coronal suture. To mathematically achieve this, the *z*‐axis coordinates for landmarks 23 and 27 were substituted with the *z*‐coordinate of landmark 20 to calculate specimen‐specific rotation angles between the original oblique plane and the new pseudo‐frontal plane. This specimen‐specific angle was then used in Equation (1) to rotate coronal suture semilandmarks, thus collapsing them into 2D. For 2D‐converted semilandmark configurations of sagittal, interfrontal and coronal sutures, see Appendix S2A–C, respectively.

(1) $$\mathrm{RM}=\left[\begin{array}{ccc} 1 & 0 & 0\\ 0 & \cos\theta & -\sin\theta \\ 0 & \sin\theta & \cos\theta \end{array}\right]$$

where, RM = rotation matrix; and θ = the angle of rotation.

Complexity metrics were implemented on the 2D semilandmarks, converted from 3D sliding semilandmarks as described above, for the three sutures (interfrontal, sagittal and coronal) for each specimen (*n* = 165). We used the two best complexity metrics identified by White et al. (2020) to quantify different aspects of suture complexity across the full comparative ontogenetic dataset (*n* = 165): Power Spectrum Density (PSD), using Fourier transforms to classify the signal of suture morphology and Fractal Dimension box counting (FD), which characterises non‐Euclidean complexity using the space‐filling properties of an object (Mandelbrott, 1982). PSD score was calculated by computing the short‐time Fourier transform (STFT), using the ‘stft’ function in the R package v. 1.7.2 (Meyer et al., 2019), after which STFT coefficients were squared over each frequency, averaged across local transforms and summed at each harmonic (Allen, 2006; White et al., 2020). FD was calculated using the ‘fd.estim.boxcount’ function in the R package *fractaldim* (Sevcikova et al., 2014). Both metrics produced a single complexity value, with higher values indicating higher complexity and lower values indicating lower complexity (Appendix S3A,B, for full and adult‐only datasets, respectively).

### 2.2 Data analysis

#### 2.2.1 Shape

To visualise the axes of shape variation across the three sutures, principal component analyses (PCAs) were performed for each suture for both the adult‐only dataset (*n* = 22) and the developmental dataset (*n* = 165), using the ‘gm.prcomp’ function in *geomorph*. MANOVAs were performed on Procrustes coordinates to assess the relative influence of developmental mode on suture shape, using the ‘procD.lm’ function in *geomorph*. For the adult‐only dataset, phylogenetic MANOVAs were performed on Procrustes coordinates with the ‘procD.pgls’ function in *geomorph*.

Allometry in suture shape was assessed across the entire dataset and for each species with a Procrustes ANOVA using the ‘procD.lm’ function in *geomorph* using Procrustes coordinates and log‐transformed centroid size. Ontogenetic allometric trajectories were computed following Morris et al. (2019) and White et al. (2025), using the ‘procD.lm’ function in *geomorph*. Pairwise ANOVAs using post‐hoc Bonferroni correction (Armstrong, 2014) were performed to assess the significance of differences between species' ontogenetic trajectories. Morphological disparity was estimated using Procrustes variances scaled by the number of semilandmarks for each suture (Zelditch et al., 2012). Procrustes variance was calculated using the ‘morphol.disparity’ function in *geomorph*, with 1000 permutations, dividing by log‐transformed centroid size to exclude allometric effects. Disparity was calculated for Procrustes coordinates for each suture using the respective mean suture shape of the dataset for each: (1) age category; (2) species (combining all developmental stages); (3) clade (combining all developmental stages); and (4) clade across age categories (Appendix S4A–C, for sagittal, interfrontal and coronal sutures, respectively).

#### 2.2.2 Complexity

To analyse ontogenetic changes in complexity for each suture, complexity scores were first regressed against the continuous proxy of developmental age (percentage of adult centroid size) using the ‘lm’ function in base R (R Core Team, 2017). ANOVAs were then performed (‘anova’ function, R package stats; R Core Team, 2017) to determine significant differences in the complexity of the three sutures across developmental stages and taxa (full dataset). A further ANOVA was performed using continuous age and developmental mode as factors for the full dataset (*n* = 165). Spearman's rank correlation was then performed to assess the relationship between suture complexity and skull size (log‐transformed centroid size from Procrustes analysis of the whole‐skull landmark data) specifying the ‘spearman’ argument within the ‘cor.test’ function in the package *stats*.

Using the adult‐only dataset (*n* = 22), phylogenetic ANOVAs were performed to test the influence of developmental mode when accounting for phylogenetic relatedness, using the ‘phylANOVA’ function in the R package *phytools* v. 0.6–0.0 (Revell, 2012). Finally, to assess evolutionary trends in suture complexity, we estimated the ancestral character state of complexity scores for each of the three sutures. To do so, we applied a maximum likelihood (ML) approach assuming Brownian motion (BM) evolutionary rates (Appendix S2, Figure S4), under the assumption that a trait may evolve via random drift as implemented by Morris et al. (2019), to the adult‐only dataset, using the ‘anc.ML’ function in *phytools* to the pruned phylogeny of Upham et al. (2019). Estimated ancestral complexities were mapped onto the branches of the tree using the ‘contMap’ function in *phytools*.

## 3 RESULTS

### 3.1 Suture shape

**FIGURE 3:**
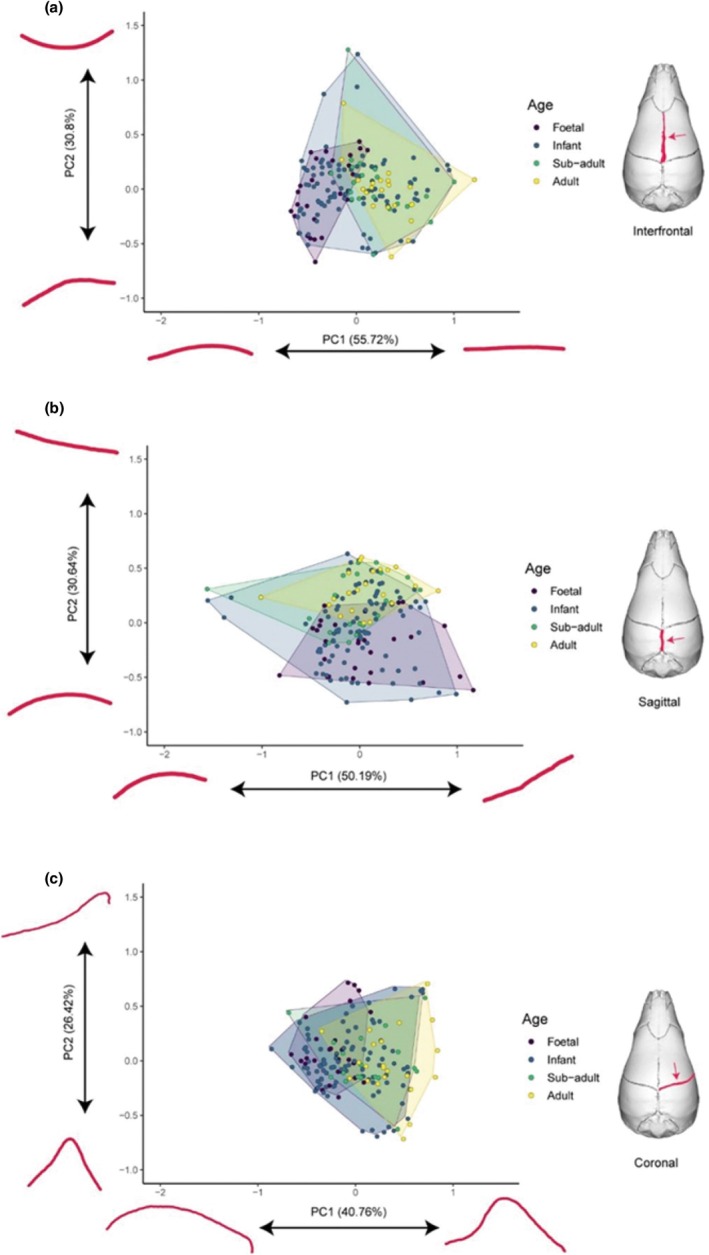
Principal component analysis of the shape of the (a) interfrontal suture, (b) sagittal suture and (c) coronal suture for the full dataset (*n* = 165). Points indicate specimens, coloured by discrete age categories and wrapped by convex hulls. Illustrated extremes of shape variation across each axis are shown in red.

Principal component analyses (PCAs) were performed separately for each suture for the full dataset (see Appendix S5A–C for the PC scores of the sagittal, interfrontal and coronal suture, respectively, for the full dataset). For the interfrontal suture, PC1 explains 55.72% of variation and captures the straightness of the suture in lateral view. PC2 explains 30.80% of the variation and reflects its dorsal convexity in lateral view (Figure 3a). For the sagittal suture, PC1 explains 50.19% of the variation and again captures the straightness of the suture in lateral view, while PC2 of the sagittal suture explains 30.64% of the variation and again reflects its convexity in lateral view (Figure 3b). For the coronal suture, PC1 explains 40.76% of the shape variation and is dominated by the curvature of the suture in dorsal view. PC2 explains 26.42% of morphological variation and is driven by the lateral position of the point of maximum flexion of the suture in dorsal view (Figure 3c).

The distribution of specimens in suture morphospace shows a clear developmental trajectory on PC1 for the interfrontal and coronal sutures, from foetal specimens on the negative end of the axis to adults on the positive end. This same distribution is also present for the sagittal suture, but on PC2 instead of PC1. For the interfrontal suture, foetal specimens occupy the smallest region of the morphospace and have the lowest morphological disparity compared to all other age categories (Procrustes variance: foetal = 0.19; infant = 0.23; subadult = 0.24; adult = 0.21), whereas the opposite is true for the sagittal suture, which also has the lowest adult disparity (Procrustes variance: foetal = 0.35; infant = 0.26; subadult = 0.29; adult = 0.25). The coronal suture instead has higher disparity values for both foetal and more prominently adult specimens compared to all other age categories (Procrustes variance: foetal = 0.28; infant = 0.26; subadult = 0.27; adult = 0.32).

No significant difference in morphological disparity is found between placentals and marsupials for the three examined sutures in both the uncorrected and the allometrically corrected analyses, as also shown by the similarity in Procrustes variances (placental vs. marsupial; sagittal: 0.28 vs. 0.27; coronal: 0.29 vs. 0.24; interfrontal: 0.23 vs. 0.21) (Table 1).

**TABLE 1:** Results of disparity tests for the three sutures across placentals and marsupials, including pairwise differences, and significance of differences between the two groups, (a) without and (b) with allometric correction.

Suture	Correction	Placental variance	Marsupial variance	Pw diff	*p*‐value
**(a)**					
Sagittal	Uncorrected	0.324	0.379	0.055	0.459
Coronal	Uncorrected	0.347	0.274	0.073	0.081
Interfrontal	Uncorrected	0.289	0.269	0.020	0.688
**(b)**					
Sagittal	Allometric	0.287	0.275	0.012	0.822
Coronal	Allometric	0.291	0.244	0.047	0.209
Interfrontal	Allometric	0.229	0.208	0.021	0.523

Although there is overlap of developmental categories in the morphospace plots, discrete age bins are a significant covariate of suture morphology (interfrontal: *R* ^2^ = 0.1341, *p* = 0.001; sagittal: *R* ^2^ = 0.0905, *p* = 0.001; coronal: *R* ^2^ = 0.1171, *p* = 0.001). Developmental mode (degree of altriciality or precociality) is also significantly associated with suture shape variation across the developmental dataset (interfrontal: *R* ^2^ = 0.2215, *p* = 0.001; sagittal: *R* ^2^ = 0.2669, *p* = 0.001; coronal: *R* ^2^ = 0.2790, *p* = 0.001).

**FIGURE 4:**
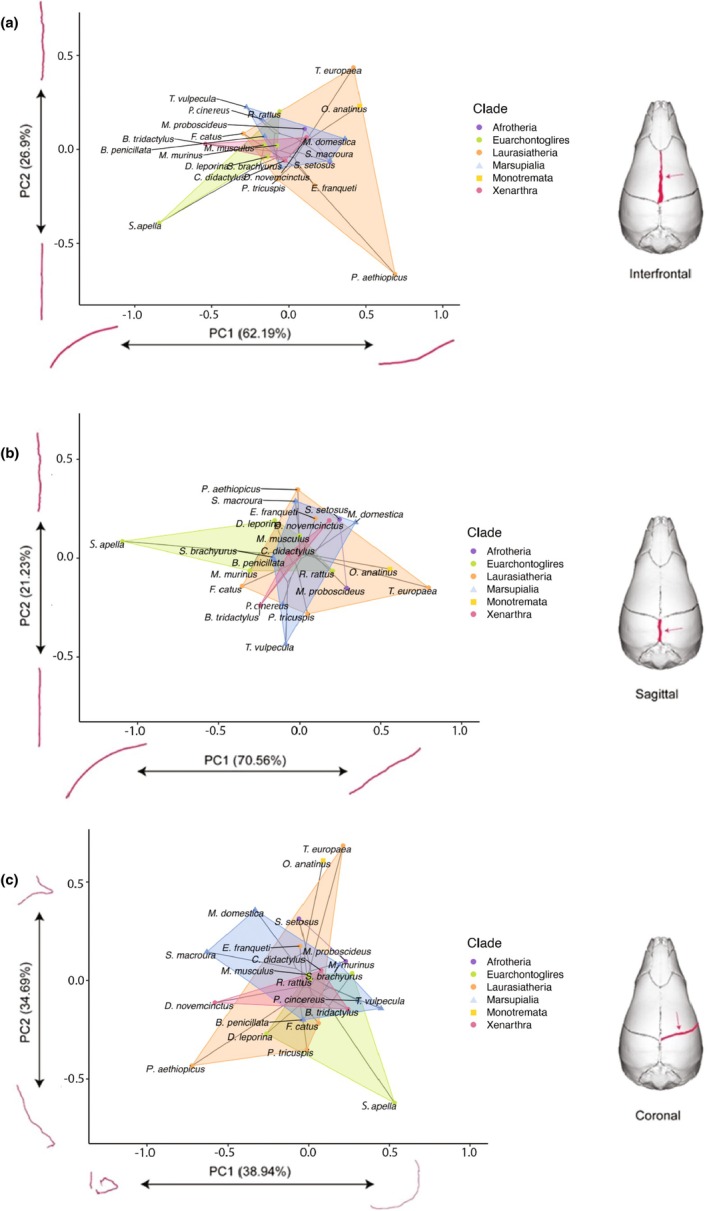
Phylomorphospace of the shape of the (a) interfrontal suture, (b) sagittal suture and (c) coronal suture for the adult‐only dataset (*n* = 22). Points indicate species averages, with shapes corresponding to mammalian subclass, coloured by clade and wrapped by convex hulls. Illustrated extremes of shape variation across each axis are shown in red.

When only adult specimens are analysed, PC1 explains 62.19% of the variation of the interfrontal suture and broadly reflects the dorsal concavity of the suture in lateral view. PC2 explains 26.9% of the variation and is dominated by the sinuosity of the suture in dorsal view (Figure 4a). For the sagittal suture, PC1 explains 70.50% of the variation and is driven by the straightness (as opposed to the dorsal convexity) of the suture in lateral view, while PC2 for the sagittal suture explains 21.23% of the variation and again reflects the sinuosity of the suture in dorsal view (Figure 4b). Lastly, for the coronal suture, PC1 explains 38.94% of the shape variation in this suture and captures the degree of three‐dimensional coiling of the suture. PC2 explains 34.69% of morphological variation and describes flexion towards the midpoint of the suture (as opposed to its lateral side) in dorsal view (Figure 4c). See Appendix S5D–F for the PC scores of the sagittal, interfrontal and coronal suture, respectively, for the adult‐only dataset.

In the adult‐only dataset, using a phylogenetically informed approach, developmental mode is significantly associated with the shape of the sagittal suture (*R* ^2^ = 0.4121, *p* = 0.005), but not with the coronal or interfrontal sutures (Appendix S6, Figure 1).

### 3.2 Ontogenetic trajectories and morphological disparity

**FIGURE 5:**
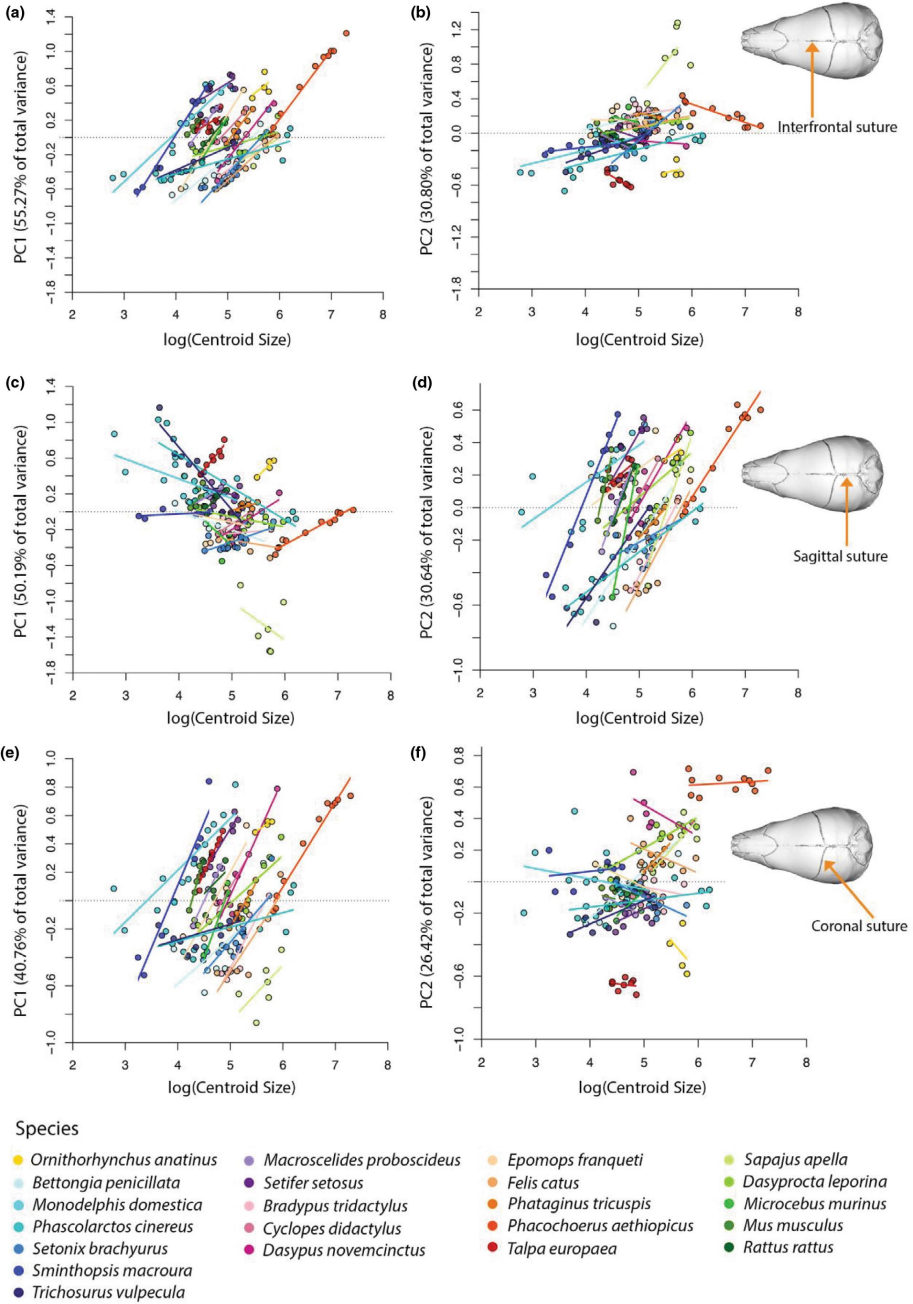
Ontogenetic trajectories of suture morphological development plotted for: (a) interfrontal suture using PC1; (b) interfrontal suture using PC2; (c) sagittal suture using PC1; (d) sagittal suture using PC2; (e) coronal suture using PC1; (f) coronal suture using PC2.

Allometry contributes significantly to suture shape across the developmental dataset (interfrontal: *R* ^2^ = 0.2084, *p* < 0.001; sagittal: *R* ^2^ = 0.1679, *p* < 0.001; coronal: *R* ^2^ = 0.1345, *p* < 0.001; Figure 5). When comparing ontogenetic allometric trajectories (morphological change through development scaled against log‐transformed centroid size) across all species included in the dataset (*N* = 22), we find a total of 79 significant differences for the coronal suture, 71 for the interfrontal and 52 for the sagittal (Appendix S6, Figure 2). Across the three sutures, differences are concentrated between marsupials and placentals, as well as in comparisons involving *Phacochoerus aethiopicus*. These analyses are multivariate across all PCs, but trajectories for PCs 1 and 2 are displayed in Figure 5 for visualisation purposes.

### 3.3 Patterns of suture complexity and associated traits

**FIGURE 6:**
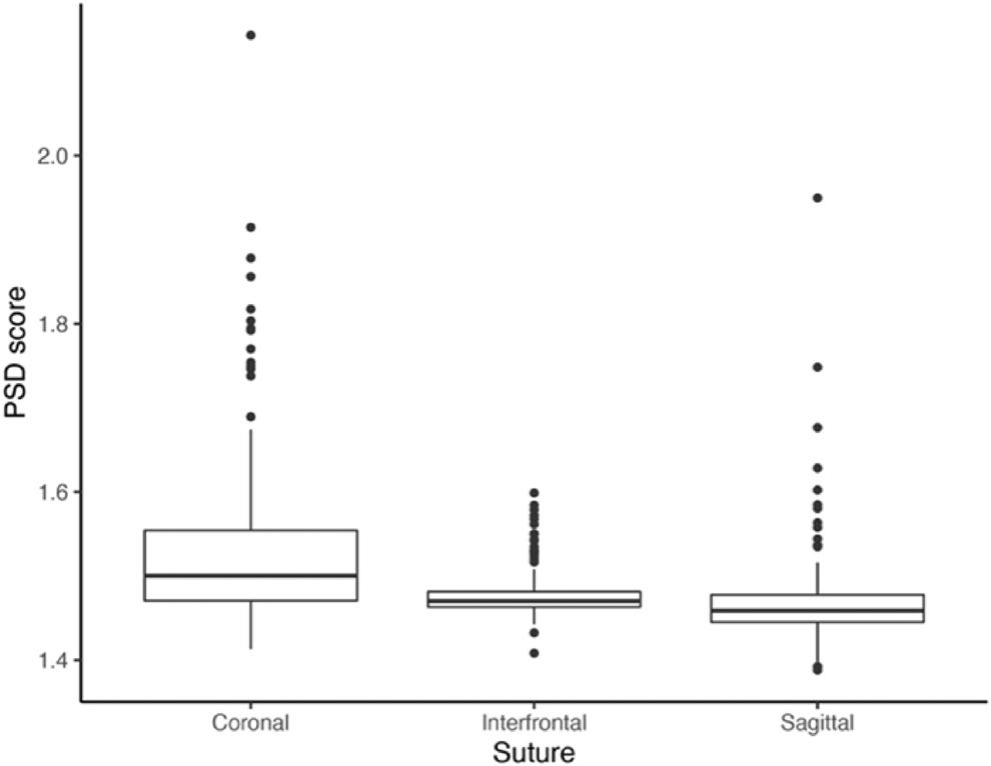
Boxplot showing the variation in suture complexity scores across the three sutures for all specimens (*n* = 165). Each boxplot indicates the median and interquartile ranges.

FD and PSD suture complexity scores (Appendix S7A) significantly correlate across the full dataset when considering all three sutures together (*p* < 0.001). When examining this relationship for each suture in isolation, significant correlations between scores are found for the sagittal (*p* = 0.005) and coronal sutures (*p* < 0.001), but not for the interfrontal (*p* = 0.855; Figure 6).

Interfrontal suture complexity ranges from 1.408 to 1.599, sagittal complexity from 1.388 to 1.950 and coronal complexity ranges from 1.413 to 2.143. Scores significantly differ across the three sutures (*p* < 0.001) and vary greatly even within the same specimen. Across the full dataset, suture complexity correlates with skull size (interfrontal: *ρ* = −0.219, *p* = 0.004; sagittal: *ρ* = −0.548, *p* < 0.001; coronal: *ρ* = −0.155, *p* = 0.046).

**FIGURE 7:**
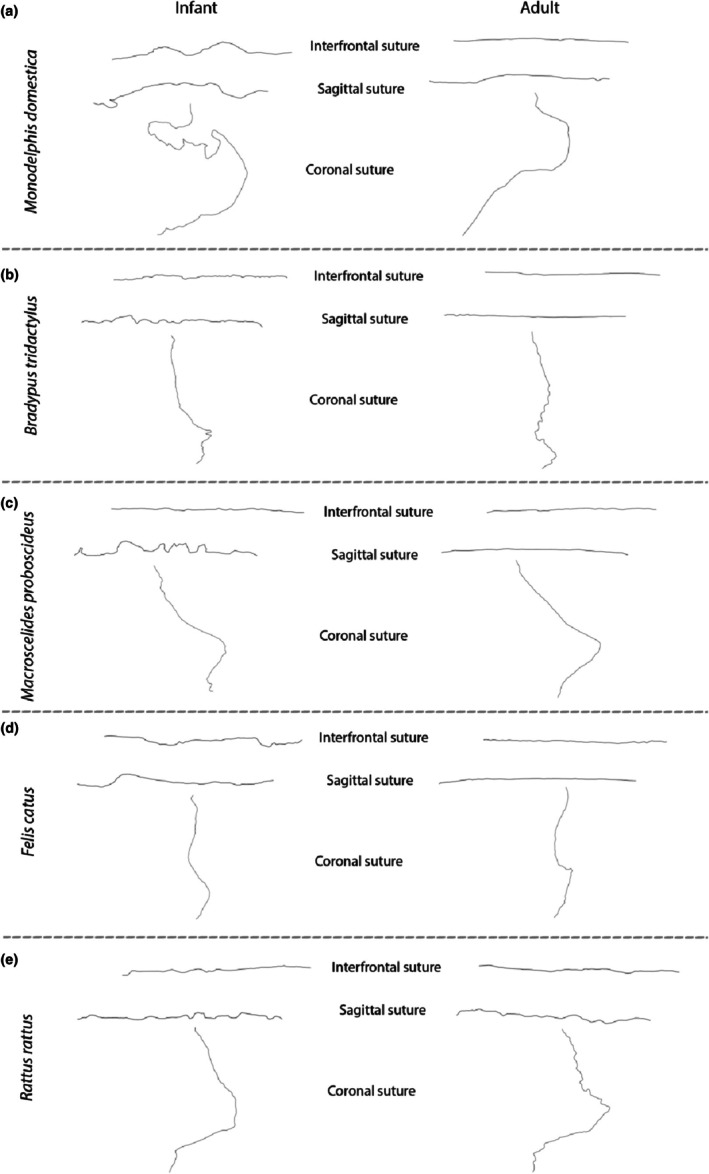
Examples of suture morphological variation at infant versus adult age stages for the following species: (a) the grey short‐tailed opossum, *Monodelphis domestica*; (b) the three‐toed sloth, *Bradypus tridactylus*; (c) the round‐eared sengi, *Macroscelides proboscideus*; (d) the common house cat, *Felis catus*; (e) the black rat, *Rattus rattus*.

Complexity decreases through ontogeny for the interfrontal and sagittal sutures (Appendix S7B), with a greater variety of complexity scores observed in early development (Figure 7). In contrast, the coronal suture shows a high diversity of complexity scores throughout ontogeny, with the greatest diversity seen in adults (Figure 7; Appendix S7B). Discrete age categories significantly correlate with complexity scores for both the interfrontal (*p* = 0.006) and sagittal sutures (*p* = 0.001), but not the coronal. For both the coronal and interfrontal sutures, marsupials display the greatest variety of complexity scores.

Developmental mode correlates with sagittal and interfrontal suture complexity (interfrontal: *p* < 0.001; sagittal: *p* = 0.004). Semi‐altricial species have the highest interfrontal suture complexity, but this is mostly driven by *Sapajus apella*. Super‐altricial and altricial species exhibit the greatest complexity scores for the sagittal suture. No clear association is found for the coronal suture.

### 3.4 Ancestral complexity

Estimated ancestral complexity scores differ between marsupials and placentals (Appendix S8: Table 1, Figure 1). We estimate a higher coronal suture complexity for the ancestral marsupial (PSD = 1.705) than for the ancestral placental (PSD = 1.532). For the interfrontal, the ancestral marsupial has a slightly lower complexity score (PSD = 1.461) than the ancestral placental (PSD = 1.474); conversely, the ancestral marsupial is found to have a slightly higher sagittal complexity (PSD = 1.457) than the ancestral placental (PSD = 1.448). For all three sutures, the ancestral therian complexity score (coronal = 1.64; interfrontal = 1.47; sagittal = 1.45) was roughly equidistant between the ancestral marsupial and ancestral placental complexity scores.

## 4 DISCUSSION

### 4.1 Variation of suture morphology across mammalia

The three sutures examined herein exhibit distinct patterns of shape and complexity across our extensive developmental dataset of mammals. Localised form‐function relationships (Gould, 2002; Lauder, 1981; Seilacher, 1970) have been ascribed to sutures (Herring, 1972), where each suture responds to different proximal functional pressures (Byron et al., 2004). Factors such as diet and behaviour are key influences on suture complexity (Buezas et al., 2017; Byron, 2009), with interdigitations resulting from strain and allowing its transmission across the skull (Byron, 2009; Herring, 1972; Herring & Teng, 2000; Jaslow, 1990; Moss, 1957). We find that the coronal suture has the highest average and maximum complexity, supporting previous studies identifying higher interdigitation in the transverse sutures than in the sutures that run antero‐posteriorly in the skull of artiodactyls (Nicolay & Vaders, 2006), rodents (Buezas et al., 2017; Sharp et al., 2023) and primates (Dzialo et al., 2014; Wang et al., 2012; Wang & Dechow, 2016). The variation observed here suggests that sutures respond locally to differing biomechanical demands (Byron et al., 2004). More specifically, suture complexity in the braincase is likely driven by signalling from the brain and dura mater via growth and transcription factors critical for suture development and the determination of suture fusion (Opperman et al., 1993, 1995; Roth et al., 1996).

### 4.2 Suture complexity and postnatal skull development in mammals

While the immediate functional aspects of sutures are an important determinant of their shape, these are necessarily mediated through developmental processes that vary throughout an animal's lifespan. Supporting this hypothesis, we identified a significant correlation between developmental mode (where a species falls on the altricial‐precocial spectrum: Grand, 1992) and suture complexity. Highly altricial and altricial species have comparably more complex sagittal‐plane sutures (the interfrontal and the sagittal) than precocial species. This contrasts with the hypothesis that precocial species would exhibit more complex sutures due to the biomechanical demands of consuming harder foods at an earlier stage of postnatal development (Starck & Ricklefs, 1998). A possible reason for this discrepancy is that the functional role of sutures as stress absorbers in mastication and locomotion (White et al., 2021, 2025) is not the only factor influencing suture complexity. In fact, this effect can be complemented by the distinct functional role as sites of interstitial bone growth while simultaneously responding to the internal pressures of the expanding brain (Lana‐Elola et al., 2007; Opperman, 2000; White et al., 2021). Altricial and super‐altricial species tend to have longer periods of postnatal brain growth and a delayed onset of full patency before adulthood is reached (Goswami et al., 2016; Smith, 2006), meaning that the effect of a developing brain is compounded with skull biomechanics, which may explain the observed higher complexities of sutures on the sagittal plane. In precocial species, both skull and brain are already significantly more developed at birth (Grand, 1992; Pagel & Harvey, 1990; Weisbecker & Goswami, 2010); thus, their skulls can be expected to undergo a lesser extent of developmental changes from infancy to adulthood, showing instead a much greater influence of biomechanical factors on suture complexity. That this difference is only detected in longitudinal sutures we examined may also indicate that tensile stress, as opposed to the compressive stress that influences the transversal sutures (Sharp et al., 2023) is less severe in precocial than in altricial species or that it has a more detectable effect on sutures likely because of the longer window of time in which they remain patent.

Placing these findings in the perspective of a developmental sequence, when we considered the trajectories of suture complexity through postnatal ontogeny in our sample, we found no consistent increase as animals age. For instance, the sagittal and interfrontal sutures are retrieved as more complex early in development, only to decrease as adulthood ensues. This decrease in complexity with increasing age contradicts the findings of previous studies (Curtis et al., 2014; Wu et al., 2007) but supports Sun et al. (2004), retrieving a similar trend for the interfrontal suture in pigs (*Sus scrofa*). By contrast, Nicolay and Vaders (2006) found an increase in suture complexity throughout ontogeny in white deer (*Odocoileus virginianus*); however, they also found that complexity plateaued at the onset of sexual maturity (>1.5 years), somewhat consistent with lowered complexity at adulthood found in our dataset. Nicolay and Vaders (2006) ascribed the observed growth‐plateau pattern to rutting behaviours in juvenile deer, due to the presence of stiffer antlers compared to adults (Blob & LaBarbera, 2001), but given that no species within our dataset have antlers, we are unable to test this hypothesis. Moreover, given that overall suture complexity does not show sex‐based differences in white‐tailed deer, rutting, a male‐specific behaviour, cannot be considered the primary cause of this decrease for that species (Nicolay & Vaders, 2006).

The reduction in complexity with age may reflect increased suture fusion in later life stages (White et al., 2025), with fusion obliterating preceding ectocranial complexity in adults. Decreasing or plateauing complexity through ontogeny has been ascribed to increased tensile loading and decreased compressive strain (Sun et al., 2004). Increased biomechanical pressure has been linked to higher ossification, leading to suture fusion and reduced complexity in adulthood (Sun et al., 2004). Moreover, increased organisation and straightening of collagen fibrils between the expanding bone fronts, which tend to reinforce skull structure as the animal ages, could also be responsible for the decrease in complexity through ontogeny (Zimmermann et al., 1998). In addition, the reaching of this complexity plateau may also correspond to a shifting necessity for pliability when the brain is still growing to a need for protection of the fully developed brain when adult stages are reached (White et al., 2021, 2025). Further, our quantification of the temporal trajectories of suture complexity may better frame the results of simulation studies examining the fit of Laplacian‐Poisson (LP) (Zollikofer & Weissmann, 2011) and reaction–diffusion (RD) models (Miura et al., 2009) to sutural systems. Across all sutures we examined, complexity exhibits nonlinear increases through morphogenesis, making them a poor predictor of developmental age and instead pointing towards local strain and morphogen distribution patterns as the likely drivers of variation (Zollikofer & Weissmann, 2011).

Like the interfrontal and sagittal sutures, the coronal suture decreases in complexity through postnatal development but then, instead of plateauing, it undergoes an increase in later postnatal stages. This refutes the picture of a common ontogenetic trajectory in complexity for all cranial sutures, or even within the cranial vault alone. Thus, complexity may not usefully predict species' developmental age, as previously proposed for suture fusion (Fujiwara & Takakuwa, 2011; Key et al., 1994; Longrich & Field, 2012; Ubelaker & Khosrowshahi, 2019). This difference in complexity trends could be explained on biomechanical grounds, as the position of coronal sutures on the skull's transverse plane as opposed to the other two sutures, which lie on the medial (sagittal) plane. Previous studies (Dzialo et al., 2014; Sharp et al., 2023; Wang & Dechow, 2016) have linked similar patterns of higher interdigitation in the horizontal sutures to the increased compressive strain of mastication, which exerts the strongest biomechanical pressures on the facial region of which the coronal suture is the posterior boundary (Shibazaki et al., 2007). In this context, our results show that, while vault sutures tend to reduce their complexity during development (likely due to the closure of skull bones: Zimmermann et al., 1998), transversal sutures of the cranial vault see increased postnatal complexity due to having a more active role in absorbing biomechanical strains such as from mastication, which arise as juveniles are weaned and start to process solid food.

### 4.3 The marsupial–placental divide or lack thereof

Comparing suture complexity across placentals and marsupials recovered surprising similarity between these two major mammalian groups. Due to the greater ecological and morphological diversity of placentals compared to extant marsupials (Bennett & Goswami, 2013; Goswami et al., 2012, 2016), the expectation was that placentals would display higher ranges of suture complexities compared to marsupials (Buezas et al., 2017; Byron, 2009; Byron et al., 2004; Herring & Teng, 2000; Jaslow & Biewener, 1995; Monteiro & Lessa, 2000). Our finding that marsupials and placentals do not show significantly different disparity in the shape of any of the sutures we examined (*p* = 0.459), nor do they differ notably in extant or estimated ancestral complexity, indicates no support for this hypothesis. Rather, marsupials in our dataset show only a marginally lower disparity in suture shape compared to placentals. Additionally, the diprotodontians in our dataset display greater variation in ontogenetic trajectories compared with placentals. The prolonged period of postnatal development (super‐altriciality) of marsupials compared to placentals (Goswami et al., 2016; Smith, 1997, 2001, 2006) may be driving the broad range of complexities we observe, especially in terms of brain growth (Weisbecker & Goswami, 2010). This longer period of development may allow marsupials to reach a level of sutural variation comparable to placentals. In contrast to placentals' more precocial brain growth, the marsupial brain primarily grows during a prolonged period of postnatal development (Smith, 1997; Weisbecker & Goswami, 2010).

Our ancestral estimations of suture complexity, while only broadly indicative of general trends, show that these inter‐group differences vary for each suture. We estimated higher complexity for the coronal suture of the ancestral marsupial (PSD = 1.7) compared with the ancestral placental (PSD = 1.53), whereas the ancestral complexity of the interfrontal (marsupials: PSD = 1.46; placentals: PSD = 1.47) and the sagittal sutures (marsupials: PSD = 1.46; placentals: PSD = 1.45) was almost identical across the two groups. The variation in the complexity of the coronal suture may reflect the different positional arrangement of the cranial bones in marsupial skulls compared to placentals (Spiekman & Werneburg, 2017), given that the coronal suture tends to be more posteriorly placed in marsupials (meaning relatively smaller parietals and bigger frontals). This also supports the hypothesised link between brain development and suture complexity (Nicolay & Vaders, 2006). As mentioned above, previous studies (Ogle et al., 2004; Slater et al., 2008) identified a link between suture morphology and the underlying dura mater meninge, which has a highly localised impact on suture morphology. Additionally, rapid brain expansion generates mechanical stress on the dura mater, influencing downstream signalling on sutures and cranium (Ogle et al., 2004; Slater et al., 2008). Therefore, this result reinforces the idea that the mechanical stress of rapid brain expansion is not the only factor that impacts suture morphology, but also prolonged brain expansion, as the action of growth itself generates stress on nearby sutures.

This overlap in extant and reconstructed ancestral suture disparities of marsupials and placentals appears to reflect our broader findings about altricial and precocial species. Specifically, it shows that the sutures of marsupials (which are mostly altricial or super‐altricial) reach comparable variation despite their purported lower ecological diversification. While the craniofacial development of marsupials is constrained by their necessity to suckle very early after birth (Goswami et al., 2016; Smith, 2006; White et al., 2025), the overall extended suture patency corresponding to a longer period of postnatal brain development may ultimately allow them to achieve similar levels of diversity in suture shape and complexity.

## 5 CONCLUSIONS

A vast variety of suture morphologies exists across species and ontogeny within Mammalia, as well as across different sutures within the same skull. This variability can be expected to reflect different ecologies and life histories, with the latter manifesting more clearly through changes during ontogeny. As species approach adulthood, contrary to previous studies (Byron, 2006; Curtis et al., 2014; Nicolay & Vaders, 2006; Wu et al., 2007), we find that suture complexity tends to plateau and then decrease in midline sutures, which we link with increased suture fusion associated with adult aging. This is true for both the sagittal and interfrontal sutures, acquiring simpler outlines by adulthood and then tending to be obliterated as the animal ages. In contrast, the coronal suture instead retains high complexity throughout ontogeny and into adulthood. This difference may be due to the different axis on which the sutures are placed, as the coronal suture's transverse position subjects it to the compressive strain of mastication (Sharp et al., 2023) as opposed to sutures that divide the skull along the midline and are subjected primarily to tensile strain.

Our study demonstrates that the greater ecological and morphological diversity of placentals compared to marsupials does not reflect in the diversity of their skull sutures. Conversely, we show that suture shape more closely mirrors cranial developmental history. In fact, contrary to existing hypotheses (Buezas et al., 2017; Byron, 2009), we find that marsupials have more varied ontogenetic trajectories compared to placentals, whilst also presenting a comparable range of suture complexities. This high complexity in marsupials may reflect their extreme altriciality and the prolonged period of postnatal brain growth that they experience. This hypothesis is further supported by the similarity we find between the suture disparities of marsupials and primates, both of which are altricial and experience long periods of postnatal brain growth (Weisbecker & Goswami, 2010, 2014). Our work thus demonstrates that suture complexity does not simply reflect the diversity of skull shapes but rather provides a distinct and informative representation of biomechanical forces—both intrinsic and extrinsic—acting upon the cranium throughout life. In addition, it shows how the trajectories of suture complexity do not follow simple patterns through the life of animals; instead, responding in different ways to a series of complex, often lineage‐specific, life‐history factors.

## AUTHOR CONTRIBUTIONS

Study design: HEW, AG; Data analysis: HEW, MC, AG; Manuscript writing and figures: HEW, MC, AG. Editing and feedback: HEW, MC, AG, AST, AW.

## CONFLICT OF INTEREST STATEMENT

The authors declare no conflict of interest.

## Supporting information


Appendix S1.

Appendix S2.

Appendix S3.

Appendix S4.

Appendix S5.

Appendix S6.

Appendix S7.

Appendix S8.

## Data Availability

All data used to conduct this research is available in the article and its supplementary materials. Code used for reproducibility can be accessed at https://github.com/Ravaat/suture_code
