# Supplementary material for: A suture in time: The ontogeny of cranial suture morphology in mammals
Source: J Anat. 2025 Aug 25;248(3):501–16. doi: 10.1111/joa.70035 (PMC12881876; doi:10.1111/joa.70035)
Supplement: Supplementary file 6 — Appendix S6. [file JOA-248-501-s006.docx]

**
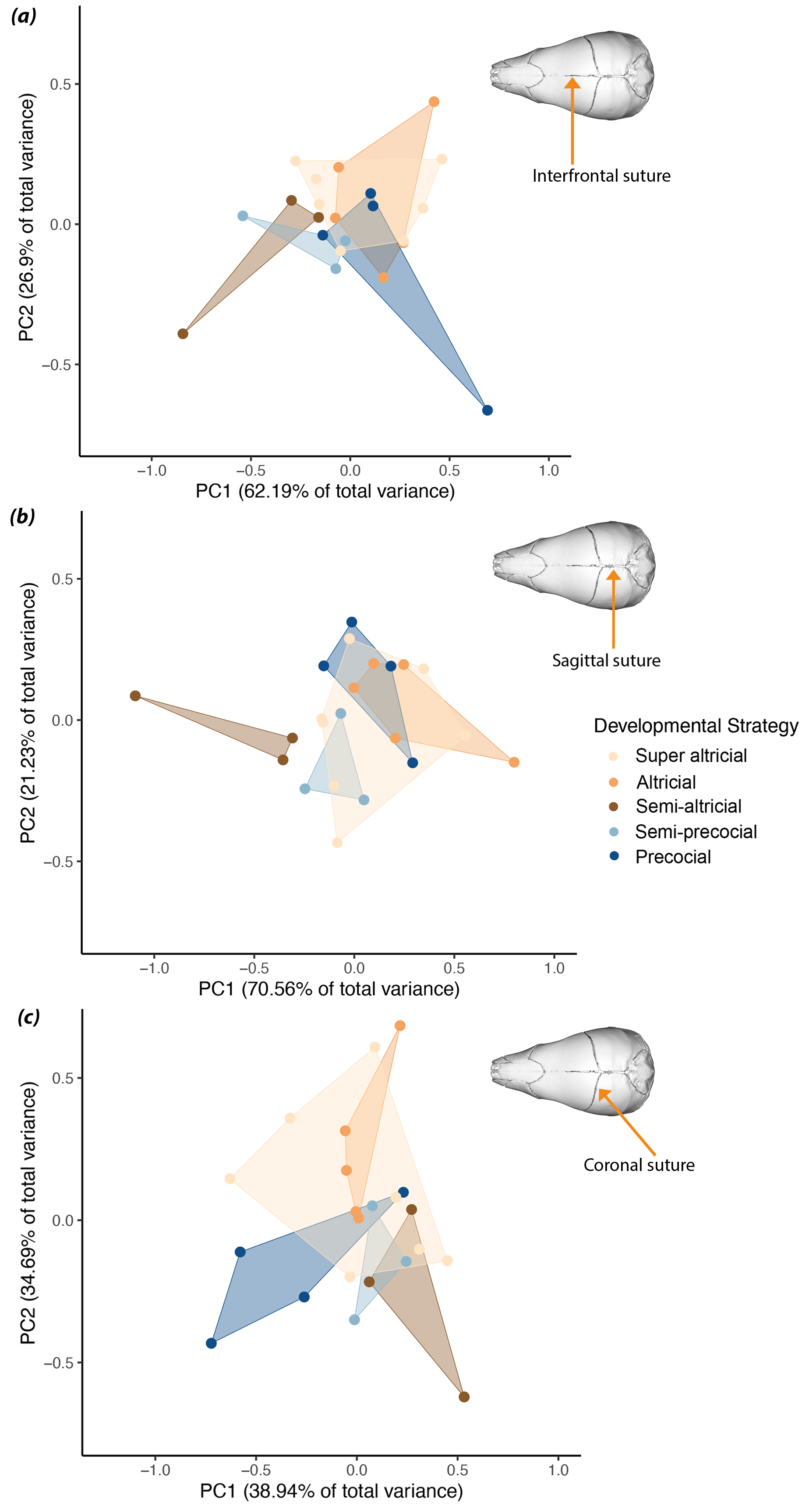
**

**Figure 1.** Morphospaces plotted with developmental strategy (indicated by colour) for the adult only dataset for the (a) interfrontal; (b) sagittal; and (c) coronal sutures.


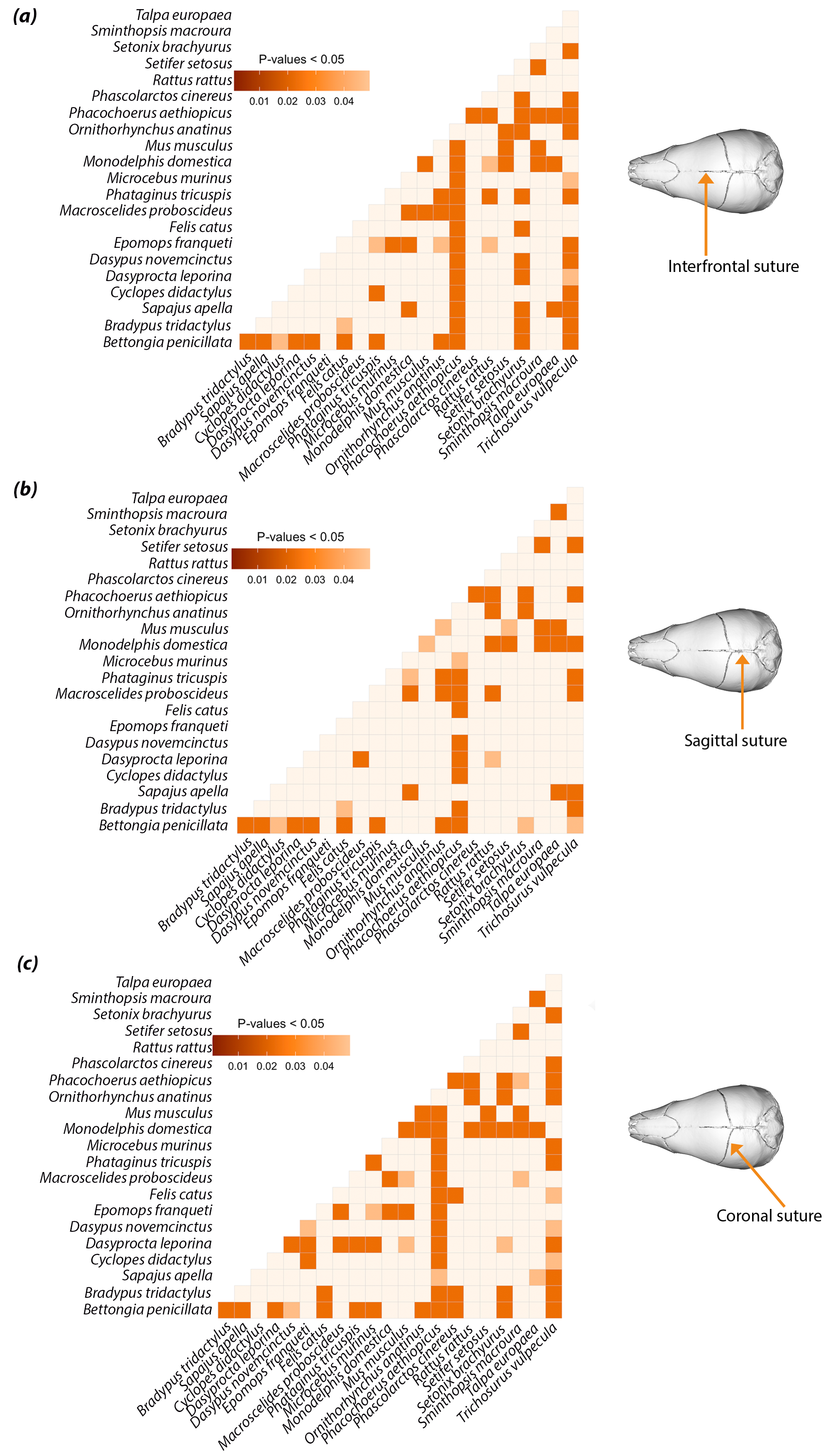


**Figure 2.** Pairwise differences across the ontogenetic trajectories of suture morphological development, for each suture analysed: (a) interfrontal; (b) sagittal; (c) coronal. Dark orange indicates significance.
