# Supplementary material for: A suture in time: The ontogeny of cranial suture morphology in mammals
Source: J Anat. 2025 Aug 25;248(3):501–16. doi: 10.1111/joa.70035 (PMC12881876; doi:10.1111/joa.70035)
Supplement: Supplementary file 8 — Appendix S8. [file JOA-248-501-s005.docx]

| **Table S14.** Reconstructed ancestral suture complexity scores for the three sutures analysed (interfrontal, sagittal, coronal), calculated using the adult specimens only.   \| Species/Ancestral node \| Interfrontal suture  PSD score \| Sagittal suture  PSD score \| Coronal suture  PSD score \| \| --- \| --- \| --- \| --- \| \| *Bettongia penicillata* \| 1.501 \| 1.398 \| 1.501 \| \| *Bradypus tridactylus* \| 1.513 \| 1.434 \| 1.513 \| \| *Sapajus apella* \| 1.487 \| 1.428 \| 1.487 \| \| *Cyclopes didactylus* \| 1.445 \| 1.451 \| 1.445 \| \| *Dasyprocta leporina* \| 1.520 \| 1.452 \| 1.520 \| \| *Dasypus novemcinctus* \| 1.477 \| 1.451 \| 1.477 \| \| *Epomops franqueti* \| 1.475 \| 1.443 \| 1.475 \| \| *Felis catus* \| 1.558 \| 1.451 \| 1.558 \| \| *Macroscelides proboscideus* \| 1.462 \| 1.471 \| 1.462 \| \| *Phataginus tricuspis* \| 1.565 \| 1.457 \| 1.565 \| \| *Microcebus murinus* \| 1.413 \| 1.432 \| 1.413 \| \| *Monodelphis domestica* \| 1.738 \| 1.468 \| 1.738 \| \| *Mus musculus* \| 1.915 \| 1.421 \| 1.915 \| \| *Ornithorhynchus anatinus* \| 1.750 \| 1.464 \| 1.750 \| \| *Phacochoerus aethiopicus* \| 1.541 \| 1.422 \| 1.541 \| \| *Phascolarctos cinereus* \| 1.475 \| 1.454 \| 1.475 \| \| *Rattus rattus* \| 1.555 \| 1.461 \| 1.555 \| \| *Setifer setosus* \| 1.528 \| 1.452 \| 1.528 \| \| *Setonix brachyurus* \| 1.597 \| 1.461 \| 1.597 \| \| *Sminthopsis macroura* \| 2.143 \| 1.476 \| 2.143 \| \| *Talpa europaea* \| 1.514 \| 1.458 \| 1.514 \| \| *Trichosurus vulpecula* \| 1.496 \| 1.445 \| 1.496 \| \| Ancestral mammal (node 23) \| 1.469 \| 1.455 \| 1.645 \| \| Ancestral therian mammal (node 24) \| 1.469 \| 1.454 \| 1.635 \| \| Ancestral placental mammal (node 25) \| 1.474 \| 1.448 \| 1.532 \| \| Ancestral Laurasiatheria (node 27) \| 1.474 \| 1.446 \| 1.533 \| \| Ancestral Euarchontoglires (node 31) \| 1.478 \| 1.444 \| 1.540 \| \| Ancestral Afrotheria (node 36) \| 1.471 \| 1.451 \| 1.521 \| \| Ancestral Xenarthra (node 37) \| 1.472 \| 1.447 \| 1.506 \| \| Ancestral Marsupialia (node 39) \| 1.461 \| 1.457 \| 1.705 \| |  |  |  |
| --- | --- | --- | --- | --- | --- | --- | --- | --- | --- | --- | --- | --- | --- | --- | --- | --- | --- | --- | --- | --- | --- | --- | --- | --- | --- | --- | --- | --- | --- | --- | --- | --- | --- | --- | --- | --- | --- | --- | --- | --- | --- | --- | --- | --- | --- | --- | --- | --- | --- | --- | --- | --- | --- | --- | --- | --- | --- | --- | --- | --- | --- | --- | --- | --- | --- | --- | --- | --- | --- | --- | --- | --- | --- | --- | --- | --- | --- | --- | --- | --- | --- | --- | --- | --- | --- | --- | --- | --- | --- | --- | --- | --- | --- | --- | --- | --- | --- | --- | --- | --- | --- | --- | --- | --- | --- | --- | --- | --- | --- | --- | --- | --- | --- | --- | --- | --- | --- | --- | --- | --- | --- | --- | --- | --- | --- | --- | --- |
|  |  |  |  |
|  |  |  |  |
| 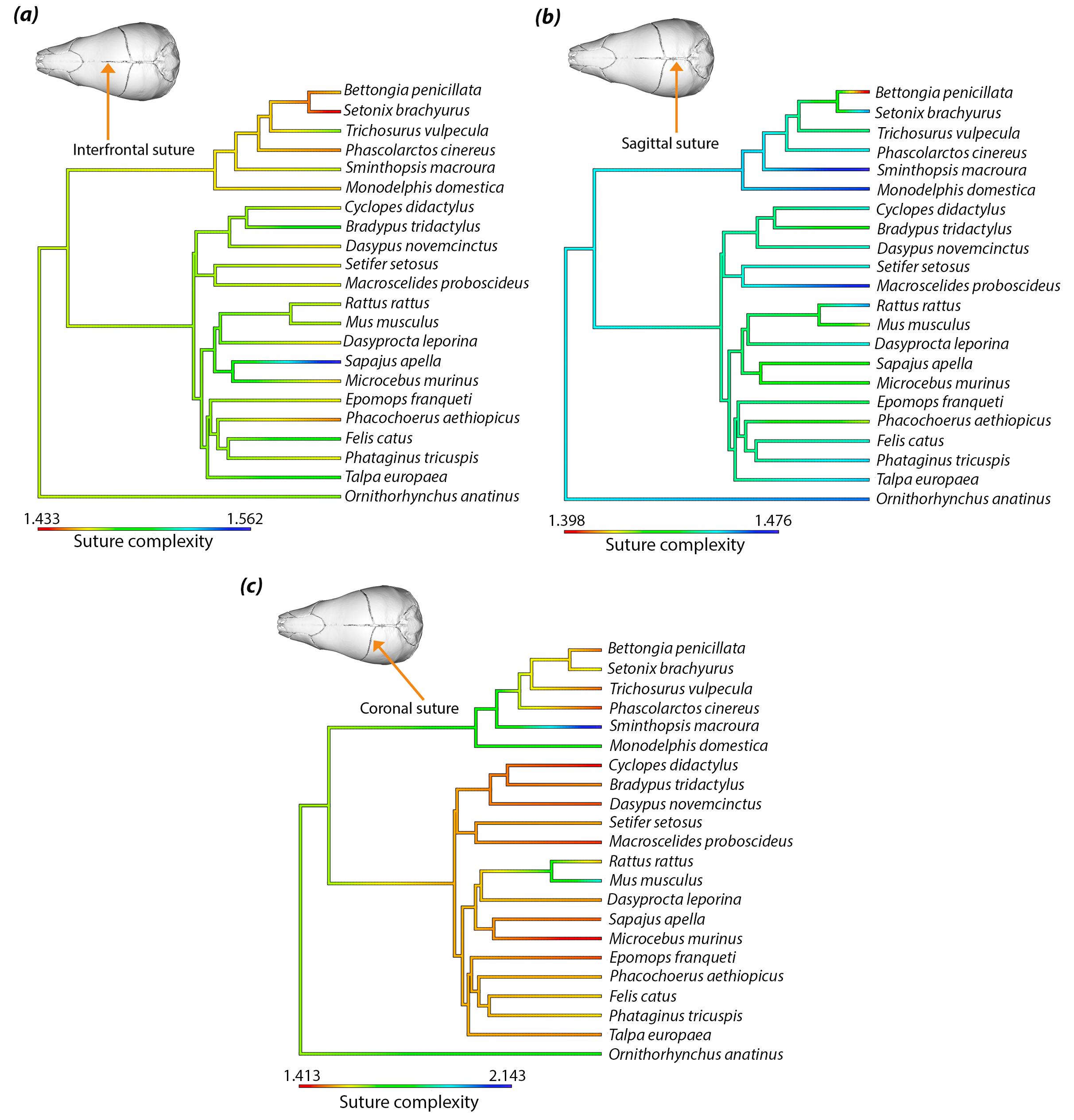  **Figure 1.** Reconstructed ancestral suture complexity scores: (a) interfrontal suture; (b) sagittal suture; (c) coronal suture. Blues indicate higher suture complexity scores, and reds indicate lower suture complexity scores. |  |  |  |
|  |  |  |  |
|  |  |  |  |
|  |  |  |  |
|  |  |  |  |
|  |  |  |  |
|  |  |  |  |
|  |  |  |  |
|  |  |  |  |
|  |  |  |  |
|  |  |  |  |
|  |  |  |  |
|  |  |  |  |
|  |  |  |  |
|  |  |  |  |
|  |  |  |  |
|  |  |  |  |
|  |  |  |  |
|  |  |  |  |
|  |  |  |  |
|  |  |  |  |
|  |  |  |  |
|  |  |  |  |
|  |  |  |  |
|  |  |  |  |
|  |  |  |  |
|  |  |  |  |
